# Targeting Tryptophan Catabolism in Ovarian Cancer to Attenuate Macrophage Infiltration and PD-L1 Expression

**DOI:** 10.1158/2767-9764.CRC-23-0513

**Published:** 2024-03-18

**Authors:** Lyndsey S. Crump, Jessica L. Floyd, Li-Wei Kuo, Miriam D. Post, Mike Bickerdike, Kathleen O'Neill, Kayla Sompel, Kimberly R. Jordan, Bradley R. Corr, Nicole Marjon, Elizabeth R. Woodruff, Jennifer K. Richer, Benjamin G. Bitler

**Affiliations:** 1 Department of Pathology, University of Colorado Denver, Anschutz Medical Campus, Aurora, Colorado.; 2 Division of Gynecologic Oncology, Department of Obstetrics and Gynecology, University of Colorado Denver, Anschutz Medical Campus, Aurora, Colorado.; 3 Antido Therapeutics, Melbourne, Australia.; 4 BioTarget Consulting, Auckland, New Zealand.; 5 Division of Reproductive Sciences Department of Obstetrics and Gynecology, University of Colorado Denver, Anschutz Medical Campus, Aurora, Colorado.; 6 Department of Immunology and Microbiology, University of Colorado Denver, Anschutz Medical Campus, Aurora, Colorado.

## Abstract

High-grade serous carcinoma (HGSC) of the fallopian tube, ovary, and peritoneum is the most common type of ovarian cancer and is predicted to be immunogenic because the presence of tumor-infiltrating lymphocytes conveys a better prognosis. However, the efficacy of immunotherapies has been limited because of the immune-suppressed tumor microenvironment (TME). Tumor metabolism and immune-suppressive metabolites directly affect immune cell function through the depletion of nutrients and activation of immune-suppressive transcriptional programs. Tryptophan (TRP) catabolism is a contributor to HGSC disease progression. Two structurally distinct rate-limiting TRP catabolizing enzymes, indoleamine 2,3-dioxygenase 1 (IDO1) and tryptophan 2,3-dioxygenase 2 (TDO2), evolved separately to catabolize TRP. IDO1/TDO2 are aberrantly expressed in carcinomas and metabolize TRP into the immune-suppressive metabolite kynurenine (KYN), which can engage the aryl hydrocarbon receptor to drive immunosuppressive transcriptional programs. To date, IDO inhibitors tested in clinical trials have had limited efficacy, but those inhibitors did not target TDO2, and we find that HGSC cell lines and clinical outcomes are more dependent on TDO2 than IDO1. To identify inflammatory HGSC cancers with poor prognosis, we stratified patient ascites samples by IL6 status, which correlates with poor prognosis. Metabolomics revealed that IL6-high patient samples had enriched KYN. TDO2 knockdown significantly inhibited HGSC growth and TRP catabolism. The orally available dual IDO1/TDO2 inhibitor, AT-0174, significantly inhibited tumor progression, reduced tumor-associated macrophages, and reduced expression of immune-suppressive proteins on immune and tumor cells. These studies demonstrate the importance of TDO2 and the therapeutic potential of AT-0174 to overcome an immune-suppressed TME.

**Significance:**

Developing strategies to improve response to chemotherapy is essential to extending disease-free intervals for patients with HGSC of the fallopian tube, ovary, and peritoneum. In this article, we demonstrate that targeting TRP catabolism, particularly with dual inhibition of TDO2 and IDO1, attenuates the immune-suppressive microenvironment and, when combined with chemotherapy, extends survival compared with chemotherapy alone.

## Introduction

Approximately 20,000 women are diagnosed with ovarian cancer in the United States annually. Five-year survival for ovarian cancer is less than 50%, an outcome that has not markedly changed in recent decades (1, 2). Epithelial ovarian carcinoma (EOC) is the most common type of ovarian cancer and has the worst prognosis, with the average 5-year survival rate for stage III/IV at a dismal 30%. The most common histotype of EOC is high-grade serous carcinoma (HGSC) of the fallopian tube, ovary, and peritoneum, and over 80% of patients with HGSC are diagnosed at an advanced stage (III/IV; refs. 3–5). The standard-of-care for HGSC is surgical debulking followed by platinum/taxane-based chemotherapies, and while about 85% of tumors initially respond, most patients will experience multiple disease recurrences culminating in therapy-resistant disease (6). Therefore, understanding and circumnavigating mechanisms of therapy resistance are essential to develop effective next-generation HGSC therapies.

HGSC has many complexities that may impact response to targeted therapies, including an immune-suppressed tumor microenvironment (TME). Immune checkpoint blockade (ICB) has emerged as a successful mechanism of inhibiting tumor growth and prolonging survival in other cancer types but has limited efficacy in successfully treating the deadliest gynecologic cancer, largely due to the poorly understood TME. To date, patients with recurrent HGSC subsequently treated with an ICB (e.g., anti-PD-L1, anti-PD1) have an objective tumor response rate of only 5% to 15%, highlighting the need to define other pathways and/or factors that affect the antitumor immune response, as well as the development of alternative or combinatorial strategies that could be employed to improve patient outcomes (reviewed in refs. 7, 8). Elevated proinflammatory cytokines, such as IL6 positively correlate with reduced progression-free survival of patients with ovarian cancer (9); however, the mechanisms of recurrence are not well understood. Better characterization of the immunogenic HGSC TME will allow actionable targeting of specific pathways to enable successful treatment with immune-based therapies.

Metabolic pathway alterations in cancer can lead to the production of cytokines and metabolites that promote tumorigenesis through the regulation of immune cells, such as regulatory T cells (Treg) and tumor-associated macrophages (TAM), as well as regulation of immune checkpoint proteins. Tryptophan (TRP) catabolism and downstream production of kynurenine (KYN) can contribute to the immune-suppressed TME (10–13). TRP is catabolized into KYN via three rate-limiting enzymes: indoleamine 2,3-dioxygenase 1 (IDO1), indoleamine 2,3-dioxygenase 2 (IDO2), and tryptophan 2,3-dioxygenase 2 (TDO2; ref. 14). KYN is a ligand for the aryl hydrocarbon (AhR) receptor, a transcription factor in tumor and immune cells that is associated with immune-suppressive effects, including altered cytokine production and expansion of Tregs and TAMs (15–17). Inhibition of IDO1 has been extensively studied in various cancers, but had limited to moderate clinical responses (reviewed in ref. 18). The functionally similar but structurally unique enzyme that catabolizes TRP, TDO2, is another promising target for preventing both the metabolism of TRP and the generation of KYN. Because of our previous data supporting the critical role of TRP catabolism in antitumor immune suppression (19), we investigated this pathway mechanistically and as a therapeutic target in ovarian cancer.

HGSC cell lines express both IDO1 and TDO2 (20). In the current study, we compared proliferation following the genetic knockdown of IDO1, TDO2, and AhR in ovarian cancer cell lines and observed a more robust antiproliferation response in the setting of TDO2 knockdown. TDO2 knockdown or pharmacologic inhibition preferentially reduced KYN levels compared with control or IDO1 knockdown or inhibition. In HGSC cells, only TDO2 overexpression (OE) was sufficient to promote growth and survival in three-dimensional (3D) cultures. Finally, dual inhibition of IDO1/TDO2 *in vivo* reduced ID8 tumor growth, macrophage infiltration, PD-L1 expression, and extended overall survival in this preclinical model.

## Materials and Methods

### Cell Lines and Culture Conditions

Human-derived OVCAR3 (RRID: CVCL_0465) and COV504 (RRID: CVCL_2424) cells were obtained from The Gynecologic Tumor and Fluid Bank at the University of Colorado (Aurora, CO). OVCAR3 cells were cultured in RPMI1640 medium (Thermo Fisher Scientific, catalog no. A4192301) plus 10% heat-inactivated FBS (Access Cell Culture) and 1% penicillin/streptomycin (Thermo Fisher Scientific, catalog no. 15140122) at 37°C and 5% CO_2_. COV504 cells were cultured in DMEM (Thermo Fisher Scientific, catalog no. A4192101) medium plus 10% heat-inactivated FBS and 1% penicillin/streptomycin. All cell lines were authenticated at the beginning of this study by short tandem repeat profiling at the Arizona Genomics Institute at the University of Arizona (Tucson, AZ; RRID: SCR_007203). Cells are monthly tested for *Mycoplasma* (LookOut, Sigma # MP0035-1KT) and were tested as recently as April 28, 2023. Cells are cultured up to 20 passages.

### Inhibitors

Epacadostat (IDO1 inhibitor, catalog no. S7190), 680c91 (TDO2 inhibitor, catalog no. S8997), StemRegenin (AhR inhibitor, catalog no. S2858), and cisplatin (catalog no. S1166) were obtained from SelleckChem. AT-0174 (IDO/TDO2 inhibitor) was provided by Antido Therapeutics, and its structure has been published previously (21). For the stability (half-life time) of the drugs, 680c91 is 16 hours (22), epacadostat is 2.4–3.9 hours (23), StemRegenin is undetermined, and AT-0174 is 4.8 hours. Drug dosages were chosen based on the provided ranges from the compound producers.

### qRT-PCR

RNA was extracted with the RNeasy Mini Kit (Qiagen, catalog no. 74004) or by TRIzol (Invitrogen, catalog no. 15596026) followed by on‐column DNase digest (Qiagen, catalog no. 79254). mRNA expression was determined using SYBR green Luna Universal One‐step RT‐PCR kit (New England Biolabs, catalog no. NEB3005) with a Bio-Rad CFX96 thermocycler. Relative gene expression was calculated using the comparative cycle threshold method, and values were normalized to β‐2‐microglobulin or 18S rRNA were used as internal controls. All primer sequences are provided in Supplementary Table S1.

### Lentiviral Transduction/TDO2 OE/Short Hairpin RNA Knockdown

Lentiviral production and transduction were performed as described previously (24). The short hairpin RNA (shRNA) IDs are listed in Supplementary Table S2. *IDO1* and *TDO2* specific shRNA (pLKO.1, RRID:Addgene_10878) and *TDO2* open reading frame (ORF; pLX-304, RRID:Addgene_25890) constructs were obtained from the University of Colorado Functional Genomics Facility. Plasmid isolation was performed using the Plasmid Midi-Prep Kit (Qiagen, catalog no. 12143). Twenty-four hours after seeding, cells were transfected with a total of 12 µg of DNA, including lentiviral packaging plasmids and the shRNA, in addition to 36 µg of polyethyenimine (PEI, Millipore, catalog no. 408719), at a ratio of 1:3 DNA:PEI. Cells were incubated overnight and transitioned to DMEM the following morning. Forty-eight hours after medium change, lentivirus was harvested. OVCAR3 cells were seeded into 6-well plates. When cells reached 80% confluence, they were transduced with lentivirus encoding gene-specific shRNAs, *TDO2* ORF, or a scrambled shRNA/empty vector (EV) control. A control well was maintained without virus to confirm puromycin selection. A 48-hour puromycin selection was performed immediately following transduction. After medium change, cells were allowed to recover then subjected to functional assays.

### *In Vitro* Growth and Survival Assays

#### Two-dimensional Proliferation Assays—Crystal Violet

A total of 5,000 cells/well were plated in a 96-well plate. After 72 hours of treatment, cells were fixed in 10% Acetic Acid/10% methanol in PBS and washed with PBS. Crystal violet (0.05% in PBS) was added to each well and then rinsed with water after staining. Plates were dried, and images were scanned for analysis.

#### Two-dimensional Proliferation Assays—IncuCyte

A total of 3,000–5,000 cells/well were plated in a 96-well plate and were then imaged for 72 hours on an IncuCyte S3 live cell imaging system at the University of Colorado Anschutz Medical Campus Cancer Center Pathology Shared Resource Cytogenetics Core Facility (RRID:SCR_021991). Cell confluence was analyzed using IncuCyte software GUI version 2019B Rev2.

#### 3D Survival Assays

As described in ref. 25, a total of 50,000 cells/well were plated in two poly-HEMA (12 mg/mL, Millipore, catalog no. P3932) coated 96-well plates. At 24 and 168 hours postseeding, cells were incubated with CellTiter-Glo Reagent (Promega, catalog no. G7570) per the manufacturer's protocol. Luminescence was measured on a Molecular Devices SpectraMax M2 or BioTek Synergy 2.0 microplate reader. Change in cell number was calculated as luminescence from day 7 divided by luminescence from day 1.

### Ovarian Cancer Dataset Analysis

Publicly available ovarian cancer datasets were interrogated, including Dependency Mapping (26). *IDO1* and *TDO2* gene effects were determined via RNAi (Achilles+DRIVE+Marcotte, DEMTER2) for the panel of cancer cell lines. RNA sequencing from The Cancer Genome Atlas (TCGA) Ovarian Serous Cystadenocarcinoma PanCancer Atlas was accessed via https://cbioportal.org (27). The mRNA expression via RSEM batch normalized from Illumina HiSeq_RNASeqV2 was evaluated for *IDO1*, *TDO2*, *AHR*, and *CD274* (PD-L1). TCGA data were accessed July 2023.

### Ultra-High Pressure Liquid Chromatography-mass Spectrometry

Global nontargeted metabolomics completed as described previously (28). Ascites fluid from patients was spun down at 1 × 1,000 × *g* for 5 minutes, and the supernatant was used for cytokine ELISA, as reported previously (15). On the basis of IL6 concentration, the 10 lowest and highest samples (total = 20) were submitted to the metabolomics resource. Global metabolomics data are reported in Supplementary Table S3. Ultra-high performance liquid chromatography-mass spectrometry metabolomics was performed by the University of Colorado School of Medicine Biological Mass Spectrometry Facility (RRID: SCR_021988). Targeted KYN and TRP mass spectrometry were performed at the Metabolomic Shared Resources at the University of Colorado (Aurora, CO; RRID: SCR_021988). Conditioned media and mouse serum were analyzed by ultra-high pressure liquid chromatography coupled to mass spectrometry as described previously (29).

### Animal Models

All animal experiments were performed in accordance with the Guide for the Care and Use of Laboratory Animals and were approved by the University of Colorado Institutional Animal Care and Use Committee (Protocol #569).

#### Tumor Study

Using the ID8 *Tp53*^−^*^/^*^−^*, Brca2*^−^*^/^*^−^ syngeneic mouse model (30), a generous gift from Dr. Ian McNeish, we examined vehicle control or AT-1074 (120 mg/kg, daily oral gavage, resuspended in 5% DMSO in sterile water) for 28 days. A total of 5 × 10^6^ ID8 cells were injected into the peritoneal cavity of 6 to 8 weeks old female C57Bl/6 mice (strain no. 000664, RRID:IMSR_000664). Tumors were allowed to establish for a week before initiating treatment. Mouse body weight was measured weekly as a surrogate for toxicity. A day before the necropsy, a subset of 4 mice per group was imaged via proton-weighted MRI at the Small Animal Imaging Shared Resource at the University of Colorado (Aurora, CO; RRID: SCR_021980). On the day of the necropsy, total tumor weight was determined, blood was collected in Ethylenediaminetetraacetic acid (EDTA)-coated tubes, and serum was isolated through centrifugation. Solid tumors were fixed in 10% neutral buffered formalin for IHC analysis.

#### Survival Study

Six to 8 weeks old C57BL/6J female mice were purchased from Jackson Laboratories (strain no. 000664, RRID:IMSR_000664) and were intraperitoneally injected with 1 × 10^6^ ID8 *Tp53*^−^*^/^*^−^ cells 7 days prior to the onset of treatment. Mice (*n* = 10/group) were treated with vehicle, cisplatin (0.5 mg/kg, weekly intraperitoneal injection, resuspended in PBS), AT-0174 (120 mg/kg, daily oral gavage, resuspended in 5% DMSO in sterile water), or cisplatin + AT-0174 for 28 days, as described. Following the cessation of treatment, we assessed overall survival in these mice. Briefly, once a mouse exhibited poor health due to excessive tumor burden (i.e., extreme distention due to ascites and/or moribundity), the mouse was euthanized, and the number of days elapsed because the cessation of treatment was recorded.

### IHC, Immunofluorescence, and Immunoblot

As described previously (24), paraffin sections were prepared for immunodetection of F4/80 (1:200, Cell Signaling Technology, catalog no. 70076, RRID:AB_2799771) and CD3 (1:200; DAKO; polyclonal). Antigens were revealed in 10 mmol/L sodium citrate dihydrate at pH 6.0 with 0.1% Tween 20 for 10 minutes at 110°C (NxGen Decloaker, Biocare). Immunodetection was performed on the Discovery Ultra autostainer (Ventana/Roche) with primary incubations for 32 minutes. Antibodies were visualized with the OmniMAP anti-Rabbit horseradish peroxidase (Roche, catalog no. 760-4311, RRID:AB_2811043) and ChromoMAP 3,3ʹ-Diaminobenzidine kit (Venata/Roche; all incubations 37°C for 12 minutes each reagent). All sections were counterstained in Harris hematoxylin for 2 minutes. Negative controls to confirm the specificity of the immunostaining included omission of the primary antibody and substitution with primary antibody diluent. IHC was performed by the University of Colorado Anschutz Medical Campus Cancer Center Pathology Shared Resource Research Histology Core Facility (RRID:SCR_021994). F4/80 and CD3 infiltrates were scored on the basis of infiltration within tumor sections by a blinded, board-certified gynecologic pathologist (M.D. Post).

For Immunofluorescence, antibodies to F4/80 (Thermo Fisher Scientific, catalog no. MF48000, RRID: AB_10376289) and PD-L1 (Cell Signaling Technology, catalog no. 64988, RRID: AB_2799672) were used in duplex with TSA Plus Cyanine 3.5 and FITC kits, respectively per manufacturer's instructions (Perkin Elmer). Slides were scanned using a Vectra 3 quantitative pathology imaging system and inForm 2.2 software was used to calculate a positive score for PD-L1 in all cells and F4/80+cells in three fields/tumor after both cell segmentation and phenotype analysis (Perkin Elmer).

Immunoblot performed as described in ref. 31. Protein was isolated in RIPA buffer supplemented with Complete EDTA-free protease inhibitors (Roche). Protein was separated via SDS-PAGE and transferred to a polyvinylidene fluoride membrane. Blots were incubated in primary antibody diluted in LI-COR TBS blocking buffer IDO1 (1:1,000, Cell Signaling Technology, catalog no. 83200, RRID:AB_2800011), AhR (1:1,000, Cell Signaling Technology, catalog no. 83200, RRID:AB_2800011), TDO2 (1:250, Abcam, catalog no. ab259359), GAPDH (1:1,000, Cell Signaling Technology, catalog no. 97166, RRID:AB_2756824), or alpha-tubulin (1:10,000, Sigma-Aldrich, catalog no. T6074, RRID:AB_477582) overnight at 4°C. Secondary goat anti-rabbit [IRDye 680RD or IRDye 800CW, LI-COR, catalog no. 92568071 (RRID: AB_2721181) or catalog no. 926-32211 (RRID: AB_621842); 1:20,000] and goat anti-mouse [IRDye 680RD or IRDye 800CW, LI-COR, catalog no. 926-68070 (RRID: AB_10956588) or catalog no. 925-32210 (RRID: AB_2687825), 1:20,000] antibodies were applied for 1 hour at room temperature. Blots were visualized using the LI-COR Odyssey Imaging System and ImageStudio software (V4). The protein expression was quantified using ImageJ software (RRID:SCR_003070).

### Multispectral IHC

Multispectral IHC (mIHC) was performed on ID8 tumors at the Human Immune Monitoring Shared Resource (RRID: SCR_021985) within the University of Colorado Human Immunology and Immunotherapy Initiative and the University of Colorado Cancer Center (P30CA046934). Antibody panel was as follows: Ly6C (1:250, Bio-Rad, catalog no. MCA2389T, RRID:AB_1102741), Arginase-1 (1:200, Cell Signaling Technology, catalog no. 93668, RRID:AB_2800207), MHC II [1:100, BioLegend, catalog no. 107601 (also 107602), RRID:AB_313316], CD206 (1:200, Cell Signaling Technology, catalog no. 24595, RRID:AB_2892682], iNOS (1:50, Thermo Fisher Scientific, catalog no. PA3-030A, RRID:AB_2152737), Ly6G [1:100, Cell Signaling Technology, catalog no. 87048 (also 87048S), RRID:AB_2909808], F4/80 (1:100, Cell Signaling Technology, catalog no. 30325, RRID:AB_2798990), WT-1 (1:100, Novus, catalog no. NB110-60011B, RRID:AB_1849479], and DAPI.

### Gynecologic Tissue and Fluid Bank

Primary human specimens were collected from patients treated at the University of Colorado Gynecologic Oncology Practice and the tissue collection protocol by approved by an Institutional Review Board (#07-935). Patients who completed written informed consent were consented in the clinic, and the primary specimens were collected on the day of surgery. Specimens were handled by the University of Colorado Cancer Center Biorepository Shared Resources and provided to the University of Colorado Gynecologic Tumor and Fluid Bank. Ascites samples from patients with high serous carcinoma were stored at −80°C. Patient information was deidentified and protected per the ethical standard of the Declaration of Helsinki (31).

### Flow Cytometry

Cells were harvested from 6-well plates at 80% confluence following the described treatment and incubation times. Approximately 120,000 cells were resuspended in 300 µL FACS buffer in 5 mL flow tubes and stained with the antibody. PD-L1-FITC [BioLegend, catalog no. 393605 (also 393606), RRID:AB_2734471] antibody were incubated for 20 minutes at 37°F in the dark. Cells were washed with 3 mL FACS buffer after incubation, resuspended in 300 µL FACS buffer, and filtered for analysis. Zombie red (BioLegend, catalog no. 423109) was used as a live/dead stain and applied to cells after filtration approximately 30 minutes before flow analysis.

The Agilent Novocyte Penteon Cytometer was used to complete all experiments. Note, the detectors on the Penteon have a dynamic range of 7.2 logarithmic decades and the response to incoming light is linear over almost the entire range. Linearity determination is an integral part of the QC Test which provides the R square values for some of the channels. Because of the broad dynamic range signals below and above 10^5^ mean fluorescence intensity are well within the linear range. All analyses were completed by trained analysts at the Flow Core at The University of Colorado Anschutz Medical Campus Cancer Center Flow Cytometry Shared Resource Core Facility (RRID:SCR_022035) and data were analyzed by the authors using FloJo. All flow experiments were conducted using nonstained controls and single stain controls. Gating was completed manually using controls and single stains. This gating strategy was applied to all samples and each sample was reviewed independently to ensure accurate gating. Baseline expression of PD-L1 was defined by the level of expression in control samples for each experiment.

### Statistical Analysis

Statistical analyses and *P*-value calculations were performed using GraphPad Prism (v9). Quantitative data are expressed as mean ± SEM unless otherwise noted. ANOVA with Tukey multicomparison correction was used to identify significant differences in multiple comparisons. *t* test was used for pairwise comparisons with FDR method of Benjamini–Hochberg. Kaplan–Meier and log-rank tests were used for survival analysis. Dose–response curves were analyzed via nonlinear regression followed by a comparison of IC_50_ using the extra sum-of-squares F Test. Multiple logistic regression with log-likelihood ratio was used to analyze patient characteristics and to assess null hypothesis. Mixed-model effects analysis was completed to determine statistical significance of *in vivo* models by determining the tumor growth (flux percent change) effect and time effect. All experiments were completed in three independent experiments and at minimum of triplicate. For all statistical analyses, the level of significance was set at 0.05. Outliers were assessed using the ROUT method with a Q value set at 1%.

### Data Availability Statement

Data are available upon request from the corresponding author.

## Results

### KYN Detection in Ovarian Cancer Patient Ascites

**FIGURE 1:**
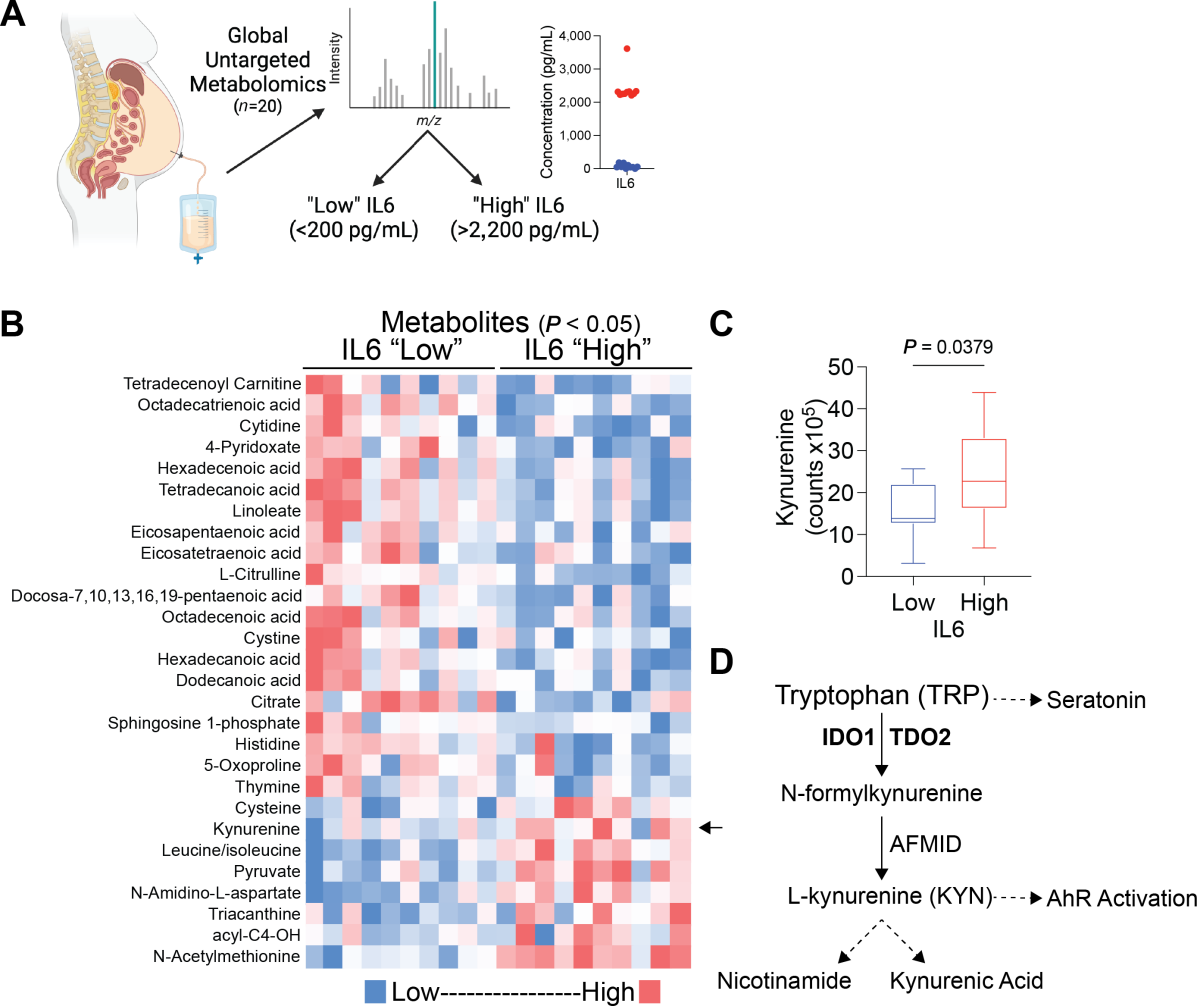
KYN enrichment in ovarian cancer patient ascites with high IL6. **A,** IL6 cytokine ELISA to stratify patients with HGSC-associated ascites. Red dots = IL6 “high” and blue dots = IL6 “low.” **B,** Global untargeted metabolomics analysis is shown from 10 patients with IL6-high and 10 with IL6-low ascites samples (*P* < 0.05). **C,** KYN levels in IL6-high and -low ascites samples with unpaired *t* test. Error bars, SEM. **D,** TRP catabolism pathway.

We began characterizing the ovarian TME using primary EOC tumor specimens and ascites from the University of Colorado Cancer Center Gynecologic Tumor and Fluid Bank (*n* = 20). We performed global untargeted metabolomics and found that IL6 was easily detectable in HGSC ascites. Because high IL6 expression in EOC correlates with a significantly shorter time to recurrence (9), we identified “low” IL6 (<200 pg/mL) and “high” IL6 (>2,200 pg/mL) groups in our cohort (Fig. 1A). IL6-high ascites had a unique metabolomics profile compared with IL6-low samples (Fig. 1B). One of the most notable differences was KYN levels, which were significantly higher in IL6-high samples compared with IL6-low (*P* = 0.0379; Fig. 1C; Supplementary Table S3). The generation of KYN from TRP is mediated by the rate-limiting enzymes IDO1 and TDO2 (Fig. 1D). Pathway analysis of enriched metabolites identified the TRP catabolism pathway as enriched in IL6-high samples (Supplementary Fig. S1).

### TDO2 and IDO Dependency in Ovarian Cancer Cells

**FIGURE 2:**
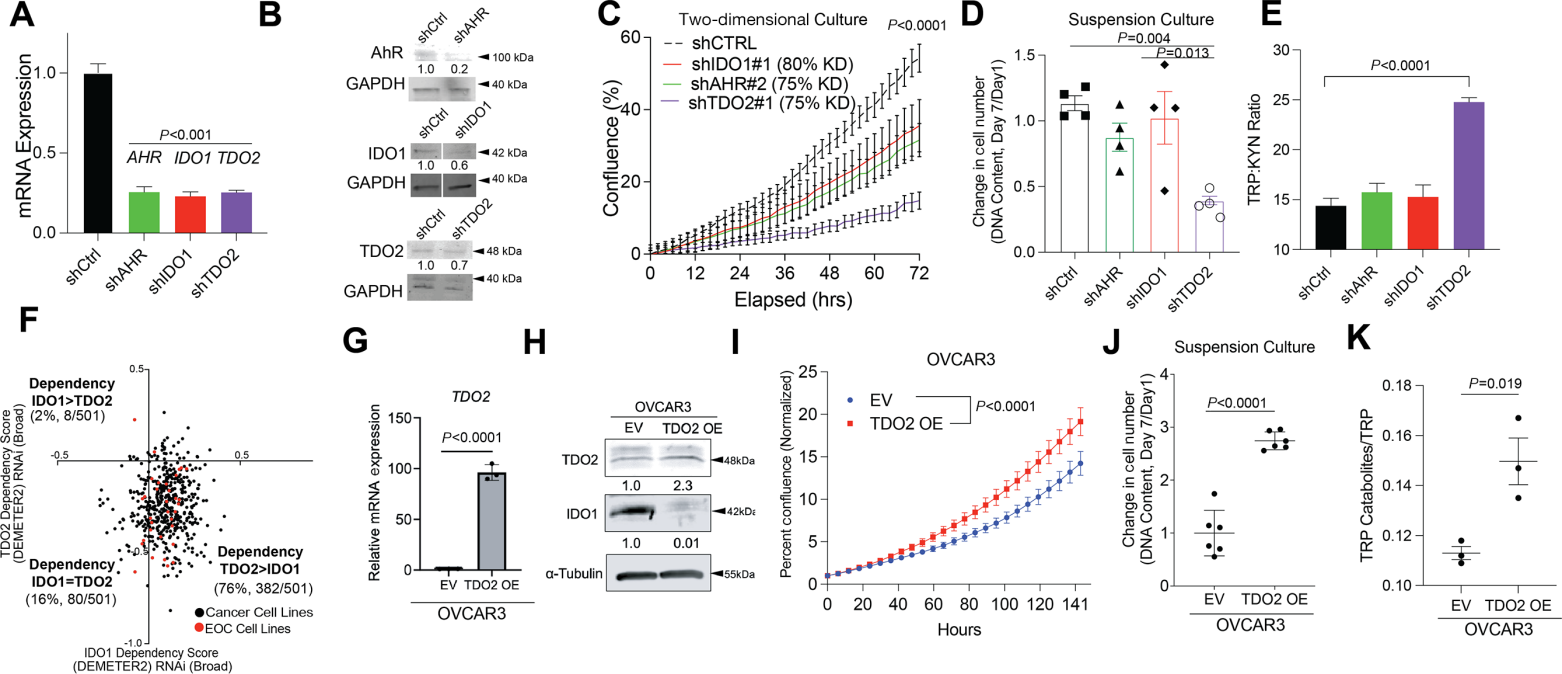
Ovarian cancer cells depend more on TDO2 than IDO1 in TRP catabolism pathway. **A,** qRT-PCR validation of OVCAR3 knockdown cells. Internal control, 18S. **B,** Immunoblot of respective knockdowns. Loading control, GAPDH. **C,** Cell confluence imaged for 72 hours. **D,** Cells cultured in suspension for 7 days. DNA content was measured on days 1 and 7. **E,** TRP and KYN levels in conditioned media from shCtrl and shKD cells over 24 hours via mass spectrometry. **F,** DepMap data (The Broad Institute) from 551 cancer cell lines. TDO2 (*y*-axis) and IDO1 (*x*-axis) dependency scores. Red dots represent EOC cell lines. Black dots represent all other cancer cell lines. **G,** EV and TDO2 OE vector transduced into OVCAR3 cells. **G,** qRT-PCR of *TDO2*. Internal control, 18S. **H,** Immunoblot of OVCAR3 EV and TDO2 OE. Loading control, alpha-tubulin. **I,** Incucyte 2D proliferation assay. **J,** 3D proliferation assay. **K,** TRP catabolites in OVCAR3 EV and TDO2 OE cells. Statistical test, unpaired *t* test (A, G, J), mixed-model effect (C, I), multicomparison two-way ANOVA (D, E).

To investigate the relative contributions of IDO1 versus TDO2, we generated shRNA-mediated knockdown IDO1 and TDO2, as well as the downstream KYN receptor AhR in human ovarian cancer OVCAR3 cells (Fig. 2A and B). Note, based on Cancer Dependency Map (DepMap) and TCGA, ovarian cancer cells do not express IDO2. Thus, we only focused on IDO1 and TDO2. *In vitro* live cell imaging to assess cell confluence revealed that TDO2 knockdown (shTDO2) cells exhibited a significant reduction in confluence over time compared with scramble controls, as well as AhR and IDO1 knockdowns (Fig. 2C), suggesting that TDO2 has the strongest effect on HGSC cell proliferation and/or cell survival. In addition, an assessment of cell growth in forced suspension culture (to mimic the detached conditions that cells must endure during metastasis) revealed that shTDO2 cells had the most robust decrease in cell number, supporting TDO2 as an important mediator of anchorage-independent cell survival (Fig. 2D). Furthermore, shTDO2 cells were the only knockdowns to alter the TRP:KYN ratio via metabolomics analysis (Fig. 2E).

To query the dependency of EOC cells on TDO2 compared with IDO1, we assessed the DepMap data of 551 cell lines, including 32 EOC cell lines and observe a higher dependency on *TDO2* compared with *IDO1*, especially in EOC cell lines (red; Fig. 2F). These data indicate that TDO2 could be a more important target than IDO in EOC. In addition, gene expression of both IDO1 and TDO2 significantly correlate with KYN in this dataset (Supplementary Fig. S2A), supporting that targeting both IDO1/TDO2 may be critical to achieve clinical efficacy.

We next tested the sufficiency of TDO2 to drive the phenotypes observed with TDO2 KD. EV and TDO2 OE OVCAR3 and COV504 cells were generated. We observed a decrease in IDO1 expression in response to TDO2 OE (Fig. 2G and H; Supplementary Fig. S2B). Live cell imaging revealed that TDO2 OE cells reached confluency faster than EV controls (Fig. 2I; Supplementary Fig. S2C). In addition, TDO2 OE cells were more viable in anchorage-independent culture (Fig. 2J; Supplementary Fig. S2D) and increased production of TRP catabolites (Fig. 2K; Supplementary Fig. S2E; Supplementary Table S4).

### Pharmacologic Inhibition of TRP Catabolism

**FIGURE 3:**
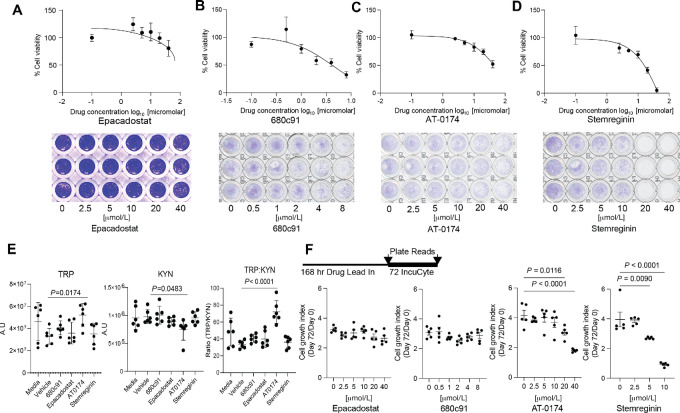
Pharmacologic dual inhibition of TDO2/IDO reduces ovarian cancer cell growth and TRP catabolism. OVCAR3 cells were treated with vehicle or increasing concentrations of epacadostat (**A**), 680c91 (**B**), AT-0174 (**C**), or StemRegenin (**D**). After 72 hours, cells were fixed and stained with crystal violet to assess viability. **E,** TRP and KYN metabolomics were performed on conditioned media from cells treated with 10 µmol/L epacadostat, 10 µmol/L 680c91, 10 µmol/L AT-0174, or 10 µmol/L StemRegenin. **F,** Cells were pretreated with increasing concentrations of epacadostat, 680c91, AT-0174, or StemRegenin for 168 hours, then cell growth over time was measured using an IncuCyte Live Cell Imaging system. Statistical test, multicomparison two-way ANOVA (E and F).

To investigate the effects of pharmacologic inhibition of individual enzymes involved in TRP catabolism, crystal violet dose–response assays using epacadostat (IDO inhibitor), 680c91 (TDO2 inhibitor), AT-0174 (IDO/TDO2 dual inhibitor), and StemRegenin (AhR inhibitor) were performed. All inhibitors reduced two-dimensional (2D) cell proliferation in OVCAR3 cells at 72 hours (Fig. 3A–D). Notably, combinational treatment of epacadostat and 680c91 showed a similar reduction trend of cell proliferation (Supplementary Fig. S3A) as AT-0174. COV504 cell viability was sensitive to pharmacologic inhibition of IDO1 alone (epacadostat), TDO2 alone (680c91), and AhR (StemRegenin 1), although surprisingly less sensitive to dual TDO2/IDO1 inhibition (AT-0174; Supplementary Fig. S3B). In OVCAR3 cells, TRP:KYN ratios were measured after 72 hours of drug treatment, and AT-0174 was the only inhibitor that demonstrated a significant increase in TRP:KYN ratio (Fig. 3E). A DepMep drug sensitivity analysis in 35 ovarian cancer cell lines revealed that treatment with 2.5 µmol/L of epacadostat over 5 days yielded highly variable responses, including induced cell growth in many cell lines such as OVCAR3 cells (Supplementary Fig. S3C). We also measured cell growth in TRP-depleted medium supplemented with exogenous KYN, and we observed that KYN alone did not rescue proliferative defects with TRP depletion, suggesting that additional TRP downstream catabolites besides KYN may also be required for TRP-mediated cell growth (Supplementary Fig. S3D). To better simulate treatment in a clinical setting with prolonged drug exposure, daily drug treatments (until 168 hours) were performed using the same inhibitors, followed by a 72-hour IncuCyte assay. AT-0174 and StemRegenin significantly reduced cell proliferation following long-term drug treatment, while epacadostat or 680c91, the monoinhibitors of IDO1 and TDO2, respectively, did not (Fig. 3F).

### Dual TDO2 and IDO1 Inhibition *In Vivo*

**FIGURE 4:**
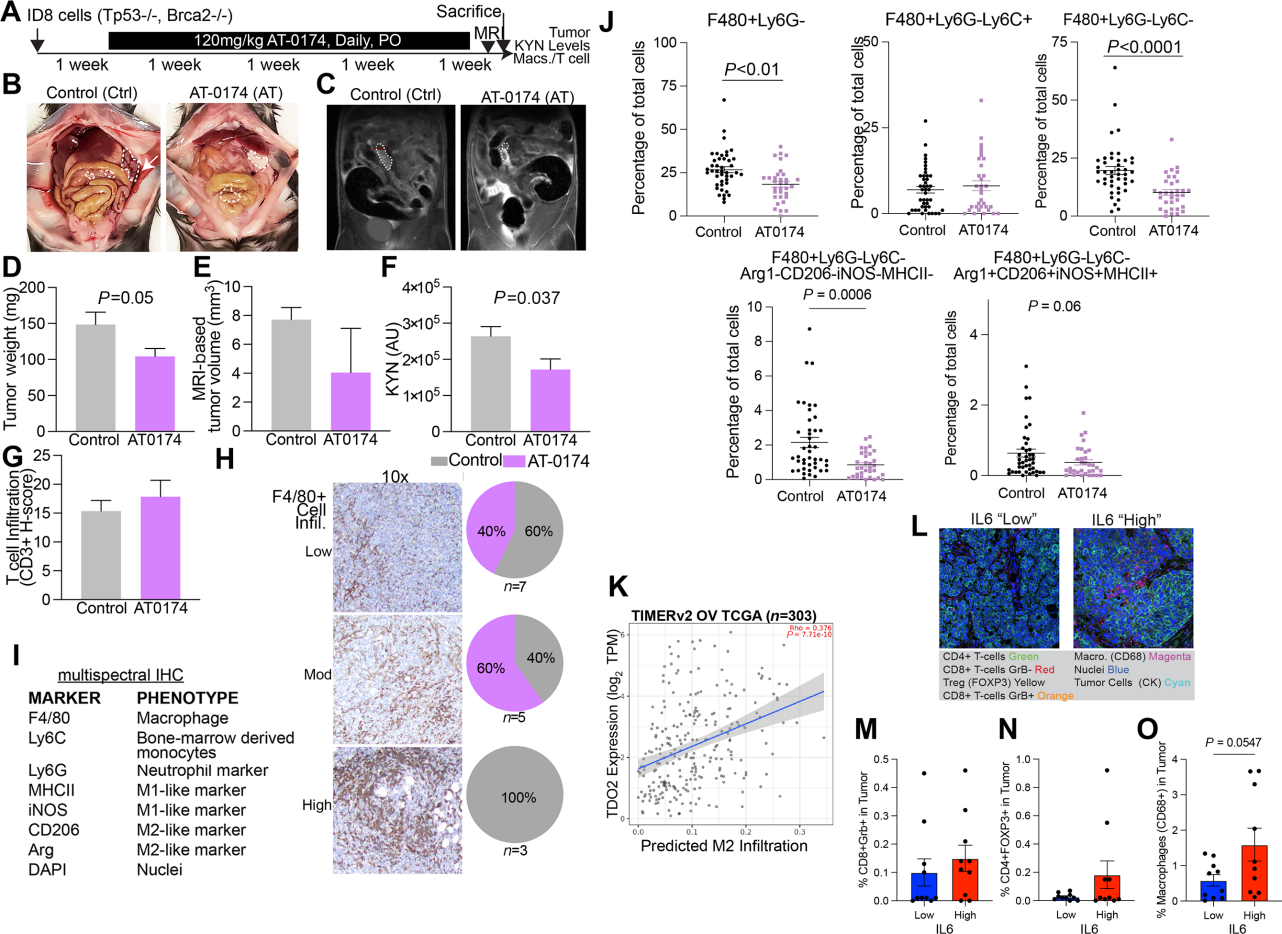
A dual IDO1/TDO2 inhibitor reduces macrophage tumor infiltration. **A,** Murine ID8 tumor study design. Representative ID8 tumor images (**B**), MRI (dotted lines; **C**), and tumor weight (*n* = 7–9/group; **D**). **E,** ID8 tumor volume. **F,** KYN levels in mouse serum. Blinded binning of T cells (CD3^+^; **G**) and macrophage (F4/80+; **H**) infiltration from ID8 tumors. **I,** mIHC panel used on ID8 mouse tumors. **J,** mIHC analysis of mouse ID8 tumors. **K,** In the human ovarian cancer TCGA dataset, TIMERv2 analysis of TDO2 and “M2” protumor macrophage infiltration. **L,** mIHC analysis of primary HGSC human tumors. Representative images. Quantification of tumor-associated cytotoxic T cells (**M**), Tregs (**N**), and macrophages (**O**) based on IL6 status of primary HGSC human tumors. Error bars, SEM. Statistical test, unpaired *t* test and unpaired *t* test with Welch correction (**M–O**). *, *P* < 0.05; **, *P* < 0.01; ****, *P* < 0.0001

We next tested the dual IDO1/TDO2 inhibitor AT-0174 in the ID8 (Tp53^−^*^/^*^−^, Brca2^−^*^/^*^−^) syngeneic HGSC model (30). AT-0174 was administered daily by oral gavage (Fig. 4A), and AT-0174 treatment significantly reduced tumor burden compared with controls (Fig. 4B–E). We verified lower KYN levels in serum of mice that received AT-0174 treatment compared with vehicle (Fig. 4F). No changes in body weight were observed in the treatment group compared with the vehicle, supporting the tolerability of AT-0174. The tumors were assessed for CD3 T-cell and F4/80 macrophage infiltration via IHC. CD3^+^ T-cell tumor infiltration was not altered by AT-0174 (Fig. 4G); however, F4/80+ TAMs were reduced in tumors of mice treated with AT-0174 (Fig. 4H). To further characterize immune alterations mediated by TDO2/KTN, we performed mIHC on the ID8 tumors from mice treated with AT-0174 or vehicle to assess whether the impacted macrophage populations were antitumor “M1-like” or protumor “M2-like” using a pan-macrophage panel that included multiple protumor and antitumor markers (Fig. 4I). The total macrophage numbers decreased with AT-0174 treatment (Fig. 4J), replicating our prior IHC data. When Ly6C stratified these populations, we found that this difference was driven by the Ly6C^−^ population, indicating that AT-0174 impacted a subset of macrophages. Surprisingly, when the protumor (Arg, CD206) and antitumor (iNOS, MHCII) states were examined using the aforementioned markers, no differences were found between the groups (Supplementary Fig. S4). However, macrophage populations that lacked all four markers (Arg1-CD206-iNOS-MHCII-) were significantly decreased with AT-0174, indicating that dual IDO/TDO2 inhibition impacts a macrophage population that is not discernable by these four polarization markers. These data suggest that TRP catabolism regulates a unique subset of TAMs. In addition, we demonstrated that elevated *TDO2* significantly correlated with high “M2-like” macrophage tumor infiltration (Fig. 4K). In human HGSC tumors, we found that IL6 expression correlates to elevated KYN levels (Fig. 1); therefore, using a previously described tissue microarray (15), we correlated the IL6 levels to the tumor immune infiltrates (Fig. 4L). Tumor-associated cytotoxic T cells (CD8^+^GranzymeB+) cells were not different compared with IL6-low and -high tumors; however, in IL6-high tumor Tregs trended up, and macrophages were significantly enriched (Fig. 4M and O). These data indicate a relationship between TDO2 and protumor macrophages and highlight a potential IL6/KYN-mediated immune suppression in primary human HGSC tumors.

**FIGURE 5:**
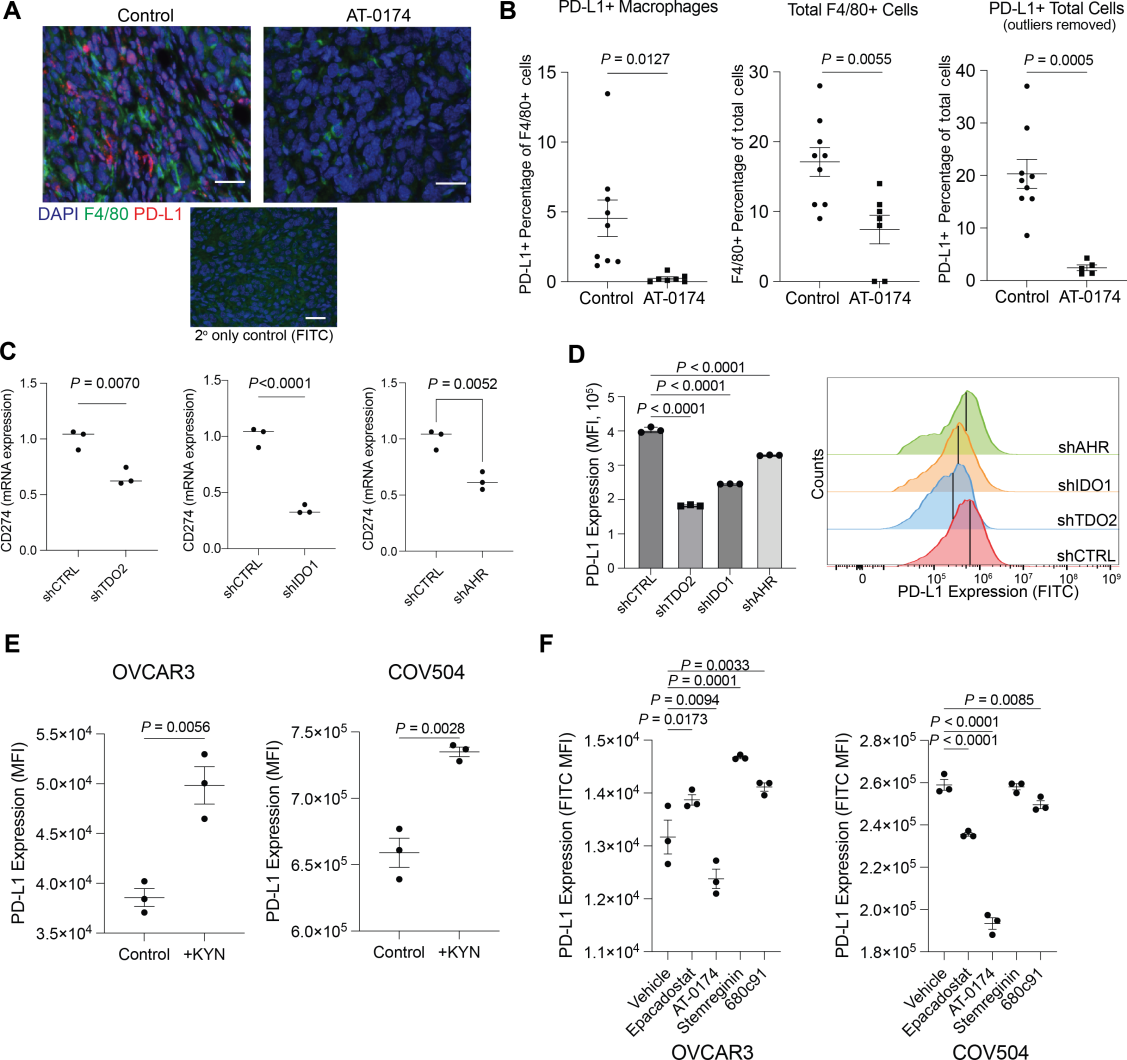
A dual IDO/TDO2 inhibitor decreases PD-L1 expression. **A,** mIHC of ID8 tumors (described in Fig. 4) for F4/80 and PD-L1. **B,** Quantification of F4/80 and/or PD-L1+ cells. **C,***CD274* (PD-L1) expression in OVCAR3 following knockdown of TDO2, IDO1, AhR. Internal controls, 18S. **D,** PD-L1 protein expression measured via flow cytometry following knockdown of TDO2, IDO1, AhR in OVCAR3. **E,** PD-L1 protein expression measured via flow cytometry following exogenous supplementation of KYN (10 µmol/L) in OVCAR3 and COV504 cells. **F,** PD-L1 expression in TDO2 or IDO inhibitor epacadostat (10 µmol/L), AT-0174 (5 µmol/L), StemRegenin (10 µmol/L) and 680c91 (10 µmol/L) treated OVCAR3 and COV504 cells. Error bars, SEM. Statistical test, unpaired *t* test.

As TDO2-induced AhR activity was recently reported to upregulate PD-L1 in colon cancer (32), we hypothesized that TDO2/KYN may promote PD-L1 expression in this unique subset of TAMs. Analysis of TCGA data revealed that *CD274* (encoding PD-L1) positively correlates with *TDO2*, *IDO1*, and *AHR* (Supplementary Fig. S5A–S5C). We then performed co-immunofluorescence for PD-L1 and F4/80 on the ID8 tumors (Fig. 5A) and found that AT-0174–treated tumors had reduced PD-L1+ macrophages, total macrophages, and total PD-L1+ cells (Fig. 5B; Supplementary Fig. S5D). These data indicate that TDO2/IDO inhibition reduces PD-L1+ macrophages, as well as PD-L1 expression on other cell types, such as tumor cells. We therefore measured *CD274* in the IDO1, TDO2, and AHR knockdown HGSC cells. *CD274* mRNA and PD-L1 protein levels were reduced in IDO1, TDO2, and AHR knockdown cells (Fig. 5C and D; Supplementary Fig. S5E). Exogenous KYN was sufficient to drive and increase in PD-L1 expression on HGSC cells (Fig. 5E). TDO2 OE was also sufficient to promote PD-L1 expression on HGSC tumor cells (Supplementary Fig. S5F). Pharmacologic inhibition of IDO1, TDO2, and AhR individually reduced PD-L1 expression, with the most robust reduction in PD-L1 observed with dual IDO1/TDO2 inhibition via AT-0174. In OVCAR3 cells, AT0174 was the only inhibitor that led to decreased PD-L1 expression. Epacadostat, StemRegenin, and 680c91 resulted in increased PD-L1 expression after treatment. (Fig. 5F; Supplementary Fig. S5G).

**FIGURE 6:**
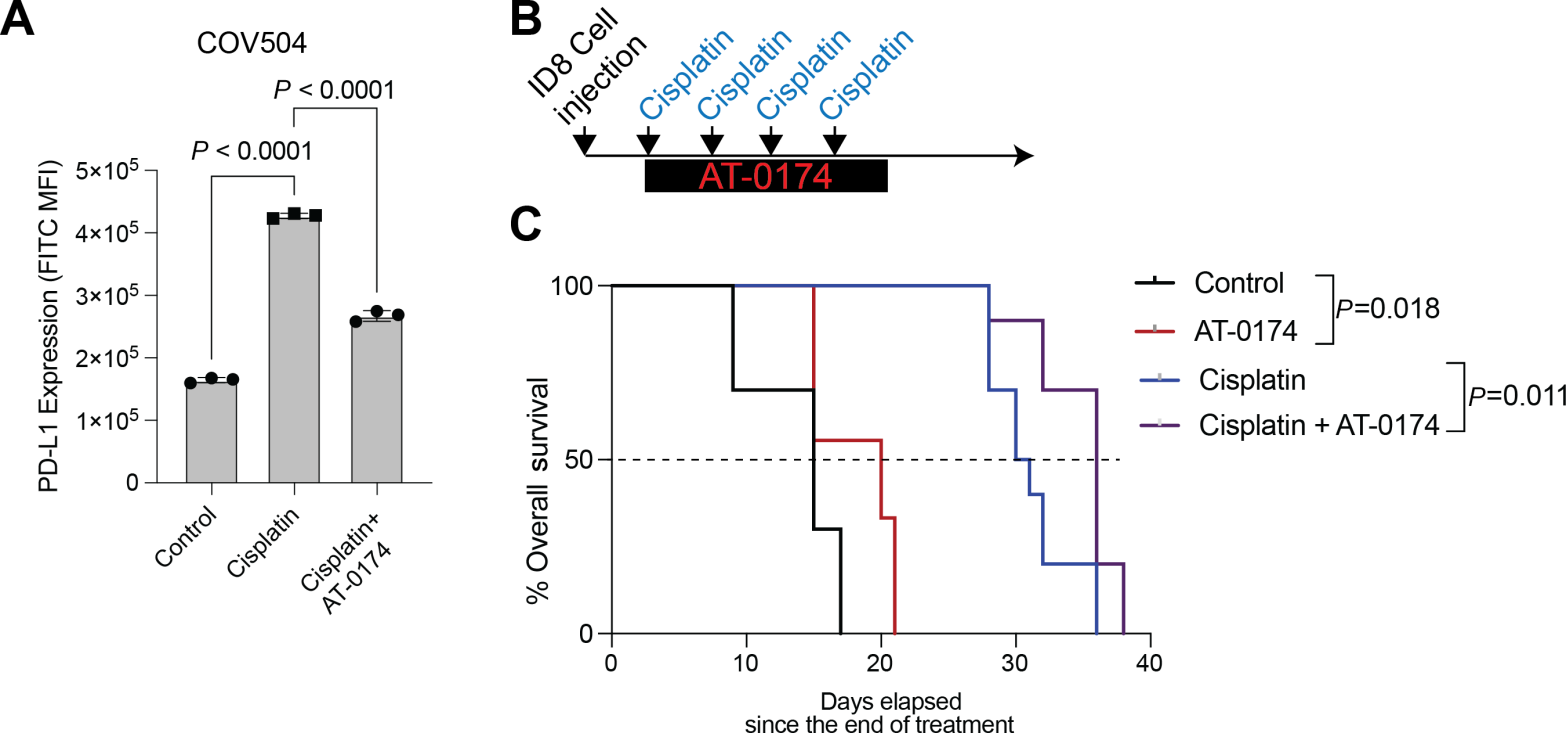
IDO1/TDO2 inhibition combined with cisplatin attenuates cisplatin-induced PD-L1 and extends overall survival. **A,** COV504 treated for 48 hours with control, cisplatin (10 µmol/L), or cisplatin (10 µmol/L)/AT-0174 (5 µmol/L). PD-L1 expression was measured via flow cytometry. **B,** ID8 (Tp53^−^*^/^*^−^) syngeneic animal study with cisplatin (i.p., weekly, 0.5 mg/kg) and/or AT-0174 (oral gavage, daily, 120 mg/kg). *n* = 10 mice/group **C**). Kaplan–Meier curve of animal study described in B. Error bars, SEM. Statistical test, multicomparison ANOVA (A) and log-rank (C).

Putting these findings into the context of HGSC standard of care (Supplementary Fig. S6A), both TRP catabolism and PD-L1 expression are elevated following exposure to platinum-based chemotherapy (33, 34). Thus, we wanted to determine whether IDO1/TDO2 inhibition attenuated chemotherapy-induced PD-L1 expression. In COV504, cisplatin treatment induced PD-L1 expression 2.57-fold (*P* < 0.0001) and the addition of AT-0174 significantly blunted the cisplatin-mediated PD-L1 induction (*P* < 0.0001; Fig. 6A). In Fig. 4, the IDO1/TDO2 inhibitor alone led to a modest tumor reduction and a significant decrease in PD-L1–expressing cells in the TME. Next, using a similar ID8 model, tumor-bearing mice were treated alone or in combination with cisplatin or AT-0174 to assess the primary outcome of overall survival (Fig. 6B). In the model, similar to a prior clinical trial with an IDO1/TDO2 inhibitor, limited toxicity was observed (Supplementary Fig. S6B). Similar to the tumor studies in Fig. 4, compared with vehicle treatment, AT-0174 treatment led to an extension of overall survival (HR: 0.436, *P* = 0.018), as did cisplatin treatment alone (HR: 0.238, *P* < 0.0001). Compared with the cisplatin-treated mice, the combination of AT-0174/cisplatin significantly improved overall survival (HR: 0.402, *P* = 0.0113; Fig. 6C). These data demonstrate that the addition of an IDO1/TDO2 inhibitor to the standard of care can extend survival in the immune-competent ID8 syngeneic HGSC model.

## Discussion

Although most patient tumors initially respond well to platinum-based chemotherapy, the disease recurs in up to 70% of patients, and the efficacy of treatment options in the recurrent setting diminishes, particularly in platinum-resistant disease. Immunotherapy checkpoint blockade has demonstrated significant clinical success in other tumor types, such as melanoma (35). However, the clinical success of checkpoint blockade against ovarian cancer as either a single agent or a combination strategy has not demonstrated meaningful clinical benefit in phase III clinical trials (36, 37). Therefore, new approaches are needed to harness the anticancer abilities of the immune system, ideally while simultaneously directly targeting tumor cells, are greatly needed. Ongoing therapeutic strategies focus on the intersection between tumor metabolism and immune suppression (38). In multiple cancer models, tumor-derived metabolites have been implicated in ICB-associated hyperprogressive disease (39). Metabolic pathways thus present a therapeutic vulnerability that can be targeted to skew the immuno-landscape to a more favorable antitumor state.

In this study, using preclinical models and clinical specimens, we determine that targeting TRP metabolism via a dual TDO2/IDO1 inhibitor reduces protumor intrinsic cell phenotypes as well as immunosuppressive markers in macrophages, which could be clinically impactful in ovarian cancer. Strategies to target IDO1 alone have been developing for years, with limited clinical trial success. In ovarian cancer, the IDO1 inhibitor epacadostat demonstrated no significant advantage over tamoxifen in a phase II clinical trial (40); however, the drug was well tolerated. In a phase I clinical trial evaluating a dual IDO1/TDO2 inhibitor in a small cohort of patients with various cancer types, treatment was well tolerated to the point that a MTD was not determined (41). Intriguingly, in that same trial, 3 patients with recurrent HGSC treated with the dual inhibitor demonstrated stable disease throughout treatment. Thus, either alone or in combination, a dual enzyme approach to targeting TRP catabolism may yield clinical benefit (41). In our current study, we observed that inhibition of IDO1 or TDO2 alone required micromolar drug concentrations to reduce cell growth, with minimal corresponding changes to TRP/KYN ratios (Fig. 3). These data suggest that higher inhibitor concentrations are needed to fully inhibit the conversion of TRP to KYN by IDO1 and TDO2, potentially due to a compensation effect between both enzymes. We also observed that ovarian cancer cells are more dependent on TDO2 than IDO1, which could further explain why single targeting of IDO1 has been clinically insufficient. Our analysis of the DepMap database revealed that low micromolar concentrations of epacadostat increased the growth of most ovarian cancer cell lines, providing additional confirmation that IDO1 inhibition alone is insufficient to reduce tumor cell growth without corresponding targeting of TDO2.

IDO1 is also reported to promote PD-L1 expression in ovarian cancer cells in an AhR-dependent manner (42). In line with this, we found that IDO1 inhibition alone (epacadostat), TDO2 inhibition alone (680c91), and dual TDO2/IDO1 inhibition (AT-0174) all reduced PD-L1 expression on ovarian tumor cells. However, unexpectedly, AhR inhibition did not affect PD-L1 expression. We postulate that the effects of TDO2 and IDO1 are mediated in part via AhR, but our data suggest an alternative mechanism of IDO1/TDO2-mediated regulation of PD-L1. It is possible that the increased KYN produced by IDO1/TDO2 activity is further catabolized into downstream catabolites that do not bind to and signal through AhR. For example, IDO1 inhibition has been reported to shift TRP catabolism toward serotonin NAD+ biosynthetic pathways (43), which could contribute to the AhR-independent function we observed. It is also possible that KYN can bind to other transcription factors in addition to AhR. The downregulation of IDO1 expression in TDO2 OE cells suggests a feedback mechanism regulating enzyme expression that warrants further attention. Future studies will investigate these possibilities.

Dual targeting of IDO1 and TDO2 may be impactful in other cancer types. Wu and colleagues recently reported that platinum-resistant non–small cell lung cancer tumors harnessed TRP catabolism via IDO1 and TDO2 to promote cell survival and immune evasion (21). They observed platinum-resistant tumors have a highly immune-suppressed TME, with tumor cell–derived KYN promoting Treg function and myeloid-derived suppressor cell activity. They also observed that the knockdown of IDO1 resulted in the upregulation of TDO2, supporting that these distinct enzymes can compensate via an uncharacterized feedback loop in the context of single inhibitors. Importantly, this first publication utilizing AT-0174, showed efficacy in preclinical models of non–small cell lung cancer, inhibiting chemoresistant tumor growth, compared with single inhibition of IDO1, supporting that TDO2 or dual inhibition is necessary for a robust antitumor response (21). This is consistent with our *in vitro* observations that knockdown of IDO1 alone was insufficient to reduce tumor cell growth in an ovarian cancer model. AT-0174, in combination with anti-PD-1 therapy, enhanced inhibition of non–small cell lung cancer tumor growth, supporting future studies of combination therapy approaches in other chemoresistant models (21).

Here we report that AT-0174 reduced intratumor levels of F4/80+Ly6G^−^Ly6C^−^ myeloid cells in an ovarian cancer model. In recurrent brain cancer, CD11b^+^Ly6G^−^Ly6C^−^ cells, termed myeloid-derived cells (MDC), are reported to stimulate pro-TAMs. MDC depletion reduced TAMs, which typically increase with chemotherapy treatment as well as therapy-associated blood vessel development (44). We, therefore, speculate that AT-0174 may affect multiple myeloid populations in ovarian cancer. Tissue-resident omental macrophages, CD163^+^Tim4+, promote tumor growth and spread in preclinical ovarian cancer murine models. Depletion of omental macrophages reduces tumor growth and ascites (45), consistent with our observations that omental macrophages are tumor promotional. Future studies will utilize macrophage depletion approaches to better characterize macrophage contribution to TDO2-driven tumor growth.

The use of AT-1074 modified the immune-suppressed TME itself and may be useful in an upfront setting for preventing changes that lead to recurrence or later resistance or in therapy-resistant recurrence settings where attenuation of the TME may permit a treatment response. Approaches combining TRP catabolism and PD-L1 inhibitors are in development in other cancer types, such as metastatic melanoma. Treatment with immunotherapy or other targeted therapies, such as MEK inhibitors, results in an increase in intratumor PD-L1 and IDO1 expression (46), suggesting recurrent tumors could be vulnerable to AT-0174. A recent phase I/II clinical trial using IO102/IO103, a first-in-class peptide vaccine targeting IDO1 and PD-L1, reported an acceptable safety profile and a median progression-free survival of 26 months (47). Importantly, as PD-L1 expression improves response to anti-PD-L1 agents and AT-0174 treatment in the HGSC models showed reduced PD-L1 expression, targeting IDO1/TDO2 in combination with anti-PD-L1 may represent a suboptimal approach. Further investigation is needed to elucidate the combinatorial approaches that exploit vulnerabilities conveyed through IDO1/TDO2 inhibition and promote a more durable treatment response. Specifically, our data show that combining IDO1/TDO2 inhibition with chemotherapy extends the overall survival of tumor-bearing mice beyond either therapy alone, highlighting a potential clinical approach to lengthening disease-free intervals.

## Supplementary Material

Supplemental Figure S1 Metabolite pathways enriched in IL6 high tumors.

Supplemental Figure S2 TDO2 overexpression alters tumor-promotional phenotypes.

Supplemental Figure S3 Pharmacologic Inhibition of TRP Catabolism.

Supplemental Figure S4 Macrophage polarization states with dual inhibition of IDO/TDO2 inhibition.

Supplemental Figure S5 Tryptophan catabolism pathway regulation of PD-L1 expression.

Supplemental Figure S6 Ovarian cancer standard of care and mouse weights

Supplementary Table 1 Supplementary Table 1. Primer Sequences

Supplementary Table 2 Supplementary Table 2. TRCN shRNA numbers

Supplementary Table 3 Supplementary Table 3. Global metabolomics in patient ascites based on IL6 expression.

Supplementary Table 4 Supplementary Table 4. TRP Catabolites in OVCAR3 EV and TDO2 Overexpression

Supplementary Methods Supplementary Methods
