# Supplementary material for: Targeting Tryptophan Catabolism in Ovarian Cancer to Attenuate Macrophage Infiltration and PD-L1 Expression
Source: Cancer Res Commun. 2024 Mar 18;4(3):822–33. doi: 10.1158/2767-9764.CRC-23-0513 (PMC10946310; doi:10.1158/2767-9764.CRC-23-0513)
Supplement: Supplemental Figure S1 — Metabolite pathways enriched in IL6 high tumors. [file crc-23-0513-s01.docx]

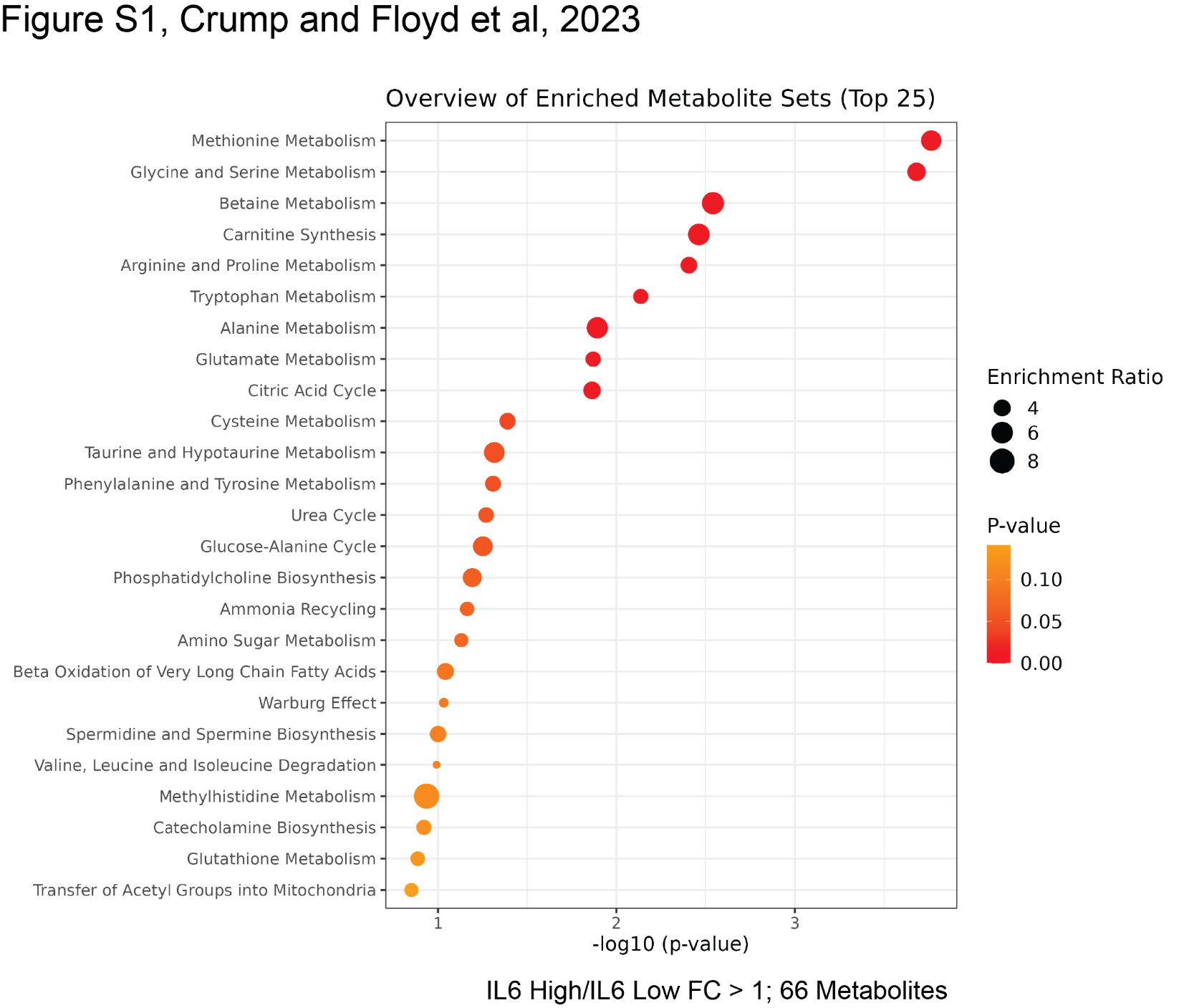


**Supplemental Figure S1. Metabolite pathways enriched in IL6 high tumors.** Pathway analysis of IL6 high vs IL6 low epithelial ovarian tumors reveals significant altered metabolic pathways, including tryptophan catabolism.
