# Supplementary material for: Targeting Tryptophan Catabolism in Ovarian Cancer to Attenuate Macrophage Infiltration and PD-L1 Expression
Source: Cancer Res Commun. 2024 Mar 18;4(3):822–33. doi: 10.1158/2767-9764.CRC-23-0513 (PMC10946310; doi:10.1158/2767-9764.CRC-23-0513)
Supplement: Supplemental Figure S2 — TDO2 overexpression alters tumor-promotional phenotypes. [file crc-23-0513-s02.docx]

**
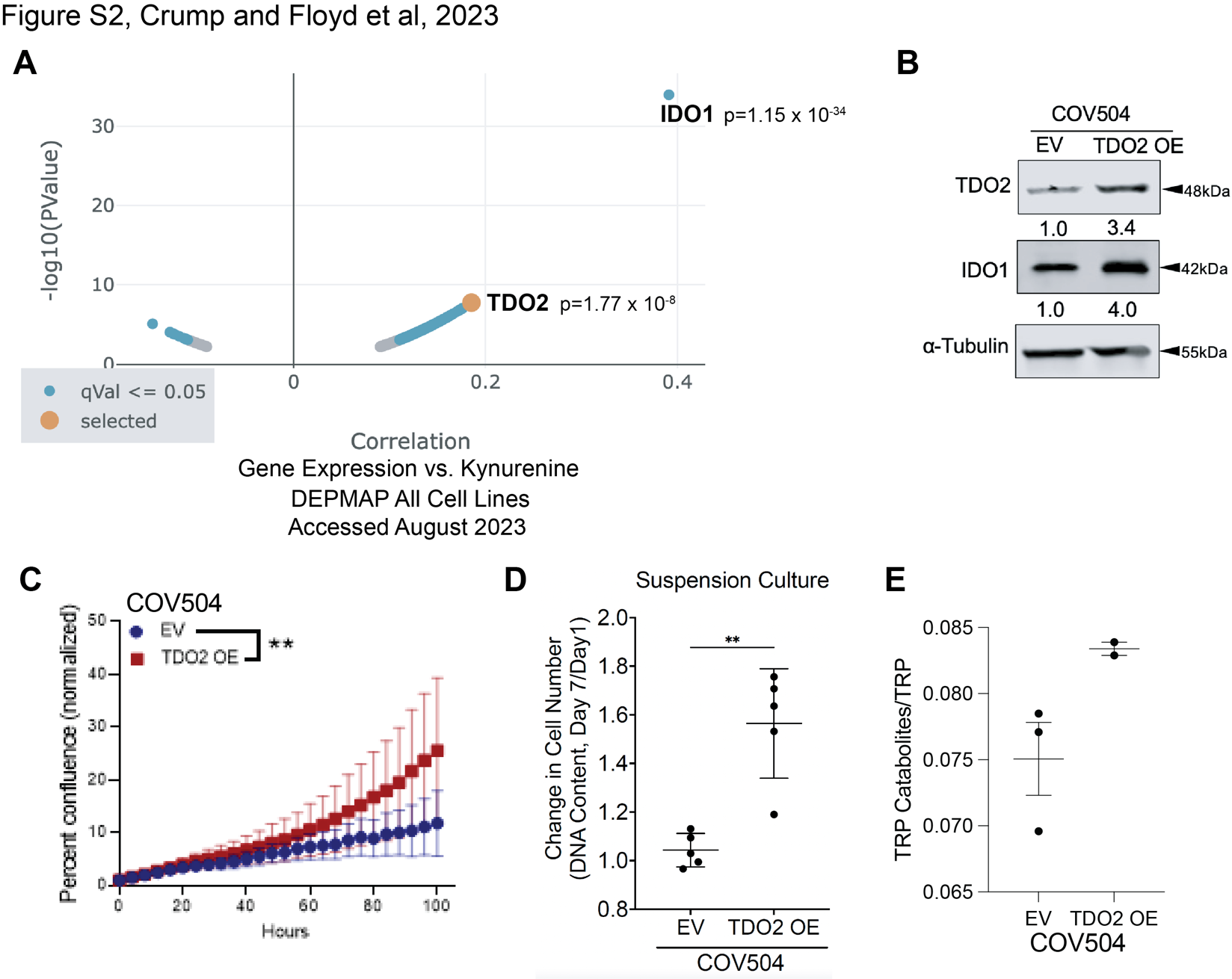
**

**Supplemental Figure S2. TDO2 overexpression alters tumor-promotional phenotypes.** A) Correlation of Kynurenine with altered gene expression in a panel of 551 cancer cell lines. Data from DepMap (The Broad Institute). B) Immunoblot validation of TDO2 overexpression (OE) compared to empty vector (EV) in COV504 cells. Loading control, α-Tubulin. C) Cell confluence imaged for 96 hrs. D) Cells cultured in suspension for 7 days. DNA content measured at day 1 and 7. E) TRP and KYN levels in conditioned media (CM) from EV and TDO2 OE cells over 24 hrs via mass spec.
