# Supplementary material for: Targeting Tryptophan Catabolism in Ovarian Cancer to Attenuate Macrophage Infiltration and PD-L1 Expression
Source: Cancer Res Commun. 2024 Mar 18;4(3):822–33. doi: 10.1158/2767-9764.CRC-23-0513 (PMC10946310; doi:10.1158/2767-9764.CRC-23-0513)
Supplement: Supplemental Figure S3 — Pharmacologic Inhibition of TRP Catabolism. [file crc-23-0513-s03.docx]

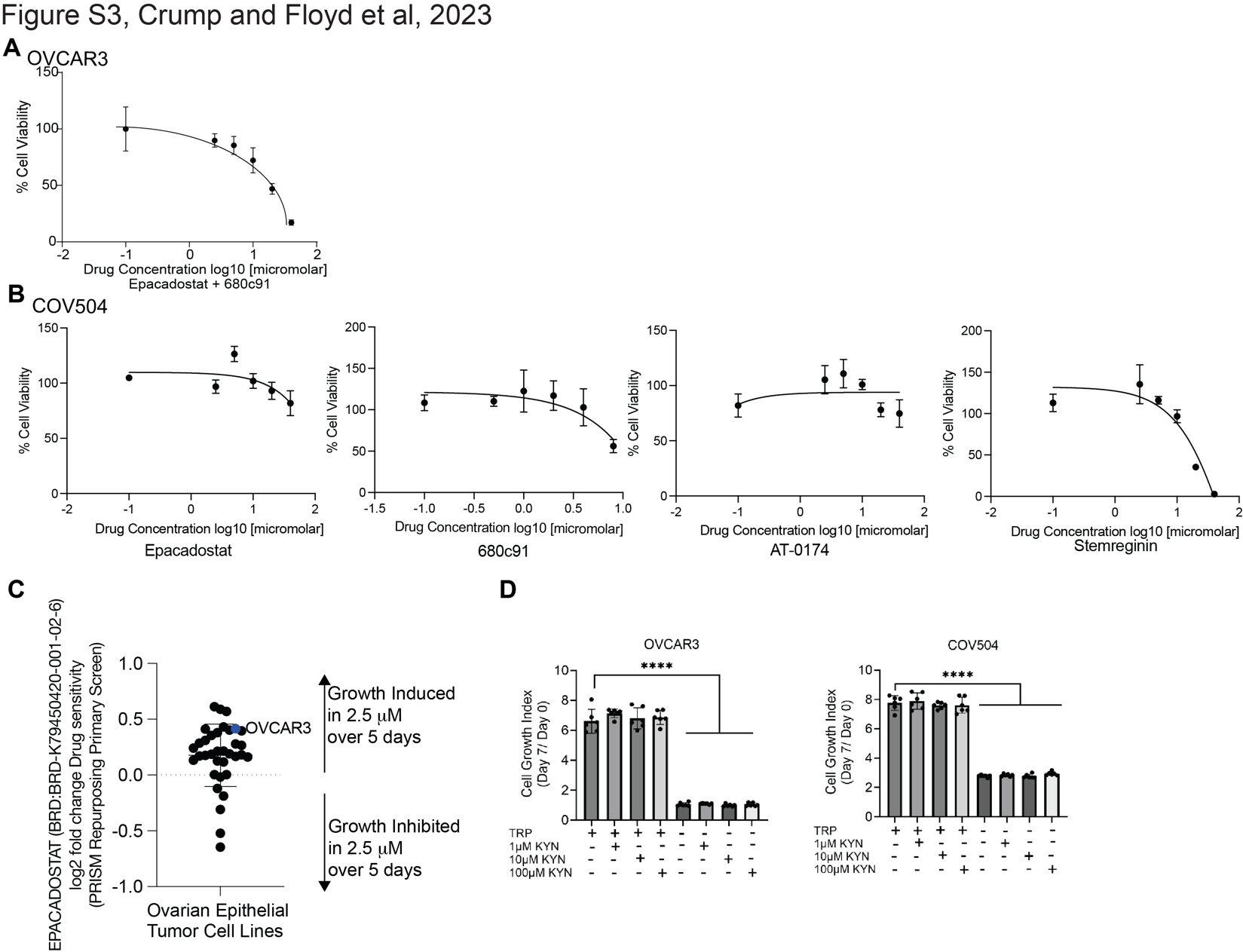


**Supplemental Figure S3. Pharmacologic Inhibition of TRP Catabolism.** **A)** OVCAR3 cells were treated with vehicle or increasing concentrations of a combination of Epacadostat and 680c91 at 1:1 (e.g., 1 μM of Epa and 1 μM of 680c91). B) COV504 cells were treated with vehicle or increasing concentrations of Epacadostat, 680c91, AT-0174, or Stemreginin. After 72 hours, cells were fixed and stained with crystal violet to assess viability. **C)** Data extracted from DepMap (accessed 02/07/24). Drug Sensitivity Data for Epacadostat in 35 Ovarian Epithelial Cell Lines treated with 2.5 μM Epacadostat for 5 days and the log2 fold change in cells is graphed. Blue dot = OVCAR3. Note: COV504 cells are not included in this dataset. **D)** OVCAR3 or COV504 were seeded in 96-well plates with RPMI1640 tryptophan containing media or tryptophan depletion media. After 8 hrs to allow cell attached, KYN was added and monitored the cell proliferation for 7 days. Error bars, SEM (A,B) and SD (C). Statistical test, one-way ANOVA with multicomparison correction.
