# Supplementary material for: Targeting Tryptophan Catabolism in Ovarian Cancer to Attenuate Macrophage Infiltration and PD-L1 Expression
Source: Cancer Res Commun. 2024 Mar 18;4(3):822–33. doi: 10.1158/2767-9764.CRC-23-0513 (PMC10946310; doi:10.1158/2767-9764.CRC-23-0513)
Supplement: Supplemental Figure S4 — Macrophage polarization states with dual inhibition of IDO/TDO2 inhibition. [file crc-23-0513-s04.docx]

**
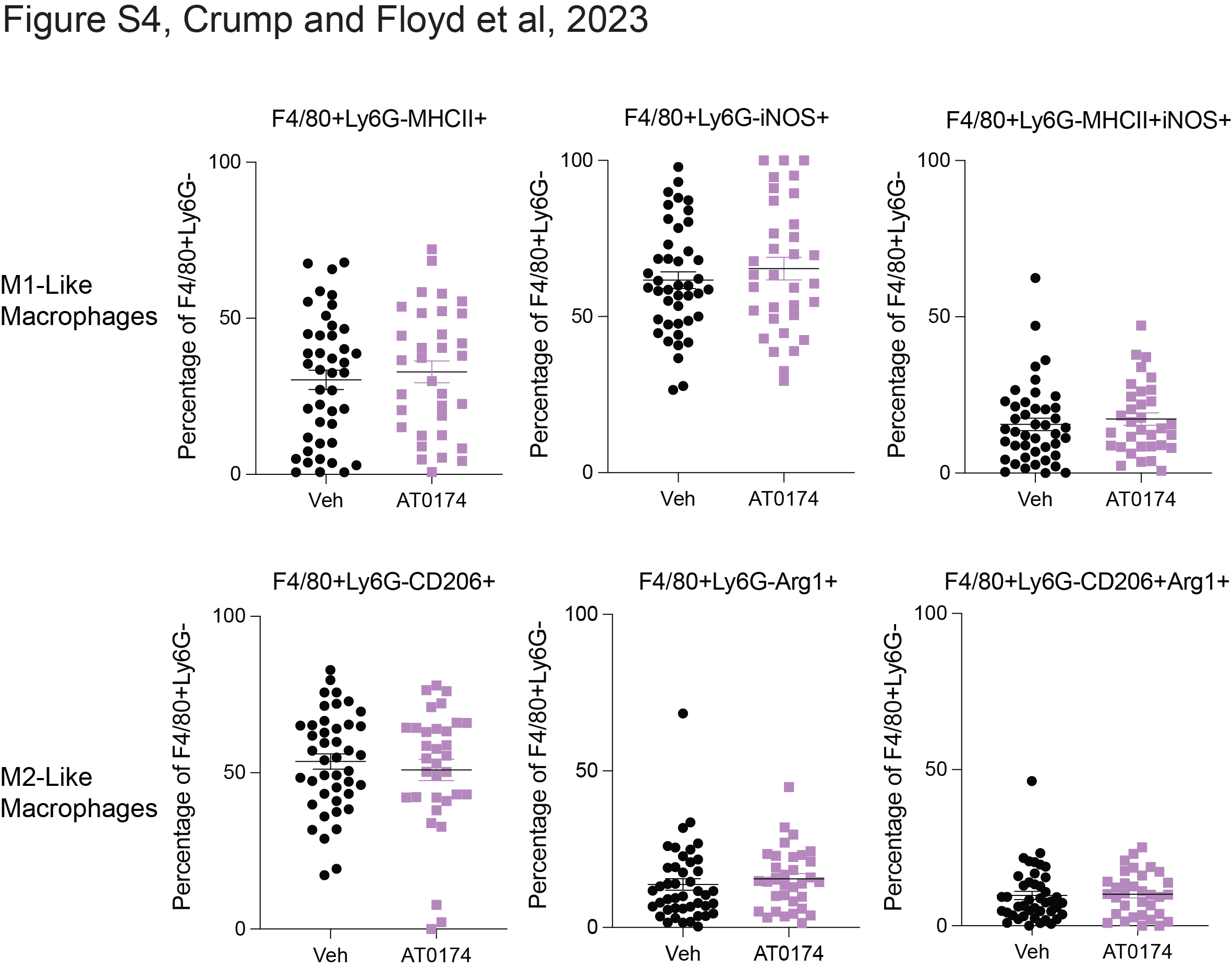
Supplemental Figure S4. Macrophage polarization states with dual inhibition of IDO/TDO2 inhibition.** Multispectral immunohistochemistry analysis of ID8 tumors, corresponding to Figure 4. Error bars, SEM.
