# Supplementary material for: Targeting Tryptophan Catabolism in Ovarian Cancer to Attenuate Macrophage Infiltration and PD-L1 Expression
Source: Cancer Res Commun. 2024 Mar 18;4(3):822–33. doi: 10.1158/2767-9764.CRC-23-0513 (PMC10946310; doi:10.1158/2767-9764.CRC-23-0513)
Supplement: Supplemental Figure S5 — Tryptophan catabolism pathway regulation of PD-L1 expression. [file crc-23-0513-s05.docx]

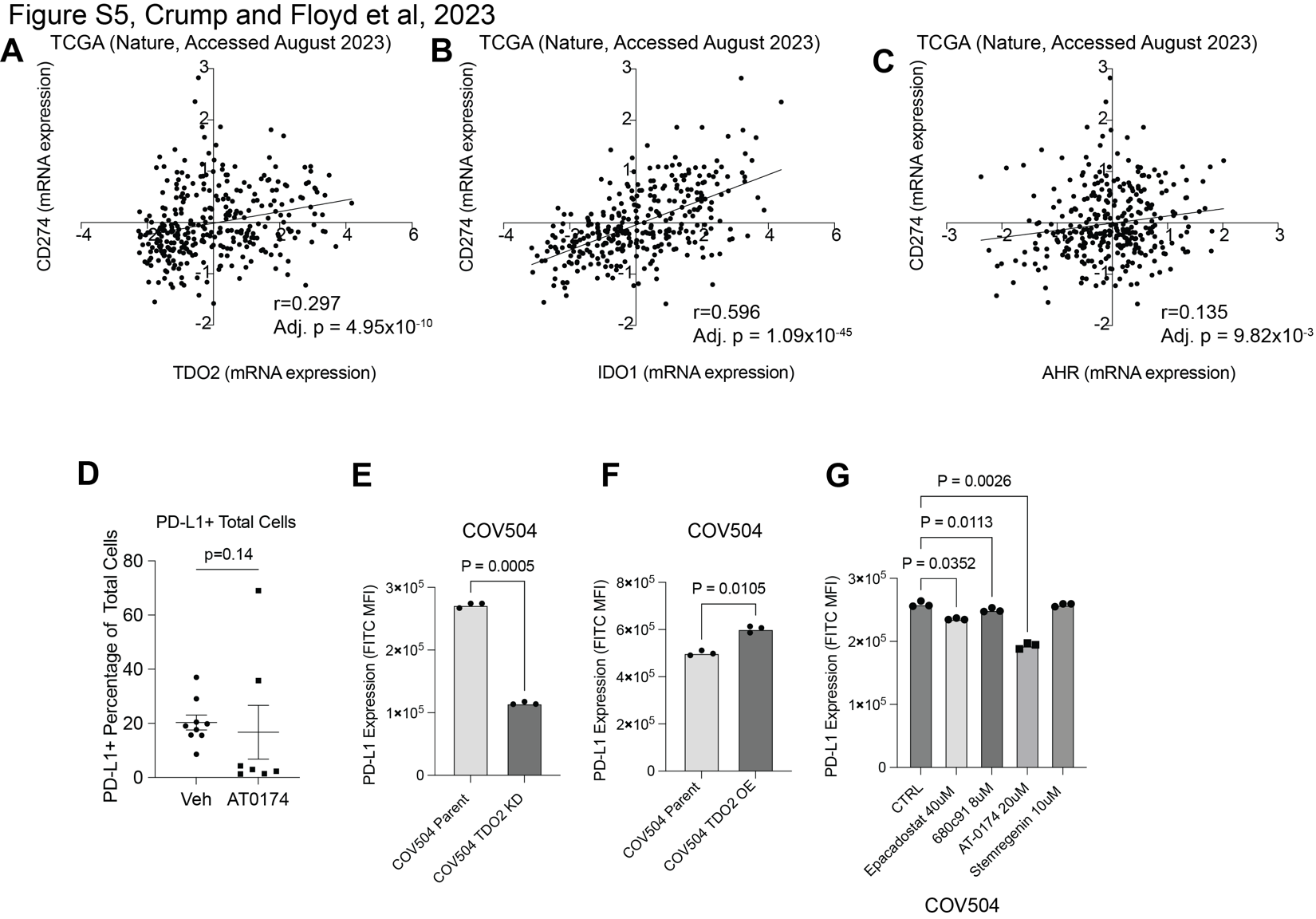


**Supplemental Figure S5. Tryptophan catabolism pathway regulation of PD-L1 expression.** The Cancer Genome Atlas (TCGA) was queried for expression of *CD274* (the gene encoding PD-L1) and A) *TDO2*, B) *IDO1*, or C) *AHR*. D) Co-immunofluorescence of F4/80 and PD-L1 in ID8 tumors, corresponding to Figure 5. E) COV504 TDO2 knockdown (KD) cells or F) TDO2 overexpression (OE) cells were assessed for PD-L1 expression via flow cytometry. G) PD-L1 expression in wildtype COV504 cells treated with inhibitors of the tryptophan catabolism pathway. Error bars, SEM. Statistical test, Spearman correlation (A-C), unpaired t-test (D-F), and one-way ANOVA with Tukey multicomparison correction (G).
