# Supplementary material for: Targeting Tryptophan Catabolism in Ovarian Cancer to Attenuate Macrophage Infiltration and PD-L1 Expression
Source: Cancer Res Commun. 2024 Mar 18;4(3):822–33. doi: 10.1158/2767-9764.CRC-23-0513 (PMC10946310; doi:10.1158/2767-9764.CRC-23-0513)
Supplement: Supplemental Figure S6 — Ovarian cancer standard of care and mouse weights [file crc-23-0513-s06.docx]

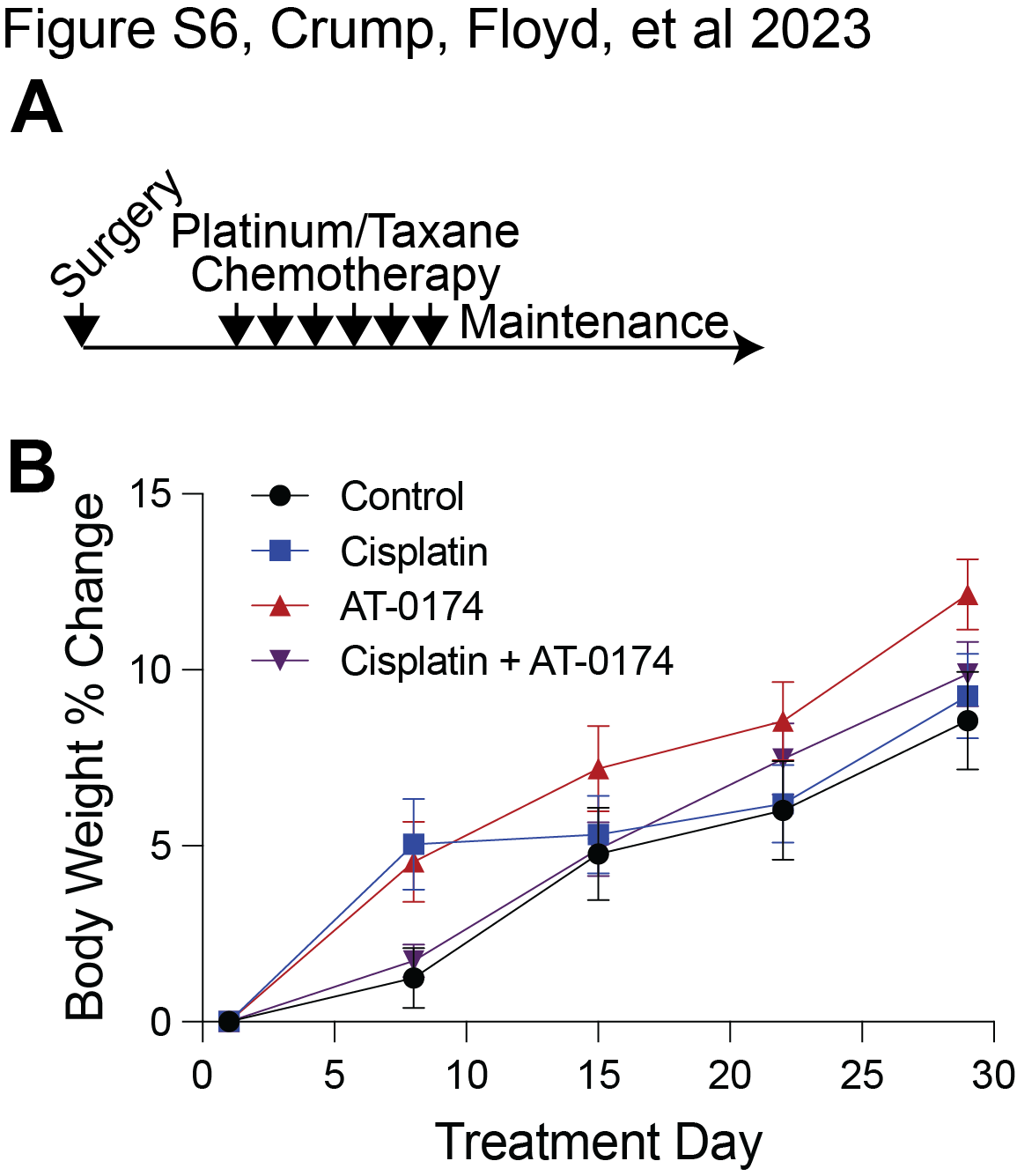


**Supplemental Figure S6.** A) Standard care timeline for High-grade serous carcinoma (HGSC) type of ovarian cancer patients. Clinical patients undergo surgery and follow platinum or taxane-based chemotherapy. B) Body weight for tumor-bearing mice during treatment used for survival studies N=10/group. Error bars, SEM.
