# Supplementary material for: Targeting Tryptophan Catabolism in Ovarian Cancer to Attenuate Macrophage Infiltration and PD-L1 Expression
Source: Cancer Res Commun. 2024 Mar 18;4(3):822–33. doi: 10.1158/2767-9764.CRC-23-0513 (PMC10946310; doi:10.1158/2767-9764.CRC-23-0513)
Supplement: Supplementary Table 1 — Primer Sequences [file crc-23-0513-s07.docx]

**Supplemental Table 1. Primer Sequences**

IDO1 – FWD – AGGATTCTTCCTGGTCTCTCT; REV - GTGTCCCGTTCTTGCATTTG

TDO2 – FWD – GTGAATAGAGCCAGCAAAGG; REV - TTGTTTCACTTTGCAGTTCTTG

AHR – FWD – CGGCATAGAGACCGACTTAATAC; REV - CGCTGAGCCTAAGAACTGAAA

CD274 – FWD – CCAGTCACCTCTGAACATGAA; REV – ATTGGTGGTGGTGGTCTTAC

18S – FWD – AACTTTCGATGGTAGTCGCCG; REV – CCTTGGATGTGGTAGCCGTTT

B2M – FWD – GGCATTCCTGAAGCTGACA; REV - CTTCAATGTCGGATGGATGAAAC
