# Supplementary material for: Targeting Tryptophan Catabolism in Ovarian Cancer to Attenuate Macrophage Infiltration and PD-L1 Expression
Source: Cancer Res Commun. 2024 Mar 18;4(3):822–33. doi: 10.1158/2767-9764.CRC-23-0513 (PMC10946310; doi:10.1158/2767-9764.CRC-23-0513)
Supplement: Supplementary Table 2 — TRCN shRNA numbers [file crc-23-0513-s08.docx]

**Supplemental Table 2. TRCN shRNA numbers**

shControl, pLKO.1, RRID:Addgene_8453

shTDO2, pLKO.1-shTDO2, TRCN0000434265

shIDO1, pLKO.1-shIDO1, TRCN0000056744

shAHR, pLKO.1-shAHR, TRCN00002452
