# Supplementary material for: Targeting Tryptophan Catabolism in Ovarian Cancer to Attenuate Macrophage Infiltration and PD-L1 Expression
Source: Cancer Res Commun. 2024 Mar 18;4(3):822–33. doi: 10.1158/2767-9764.CRC-23-0513 (PMC10946310; doi:10.1158/2767-9764.CRC-23-0513)
Supplement: Supplementary Table 4 — TRP Catabolites in OVCAR3 EV and TDO2 Overexpression [file crc-23-0513-s09.docx]

**Supplemental Table 4. TRP Catabolites in OVCAR3 EV and TDO2 Overexpression**

|  |  | **EV** | **EV** | **EV** | **TDO2 OE** | **TDO2 OE** | **TDO2 OE** |
| --- | --- | --- | --- | --- | --- | --- | --- |
| **compound** | **CmpdID** | **1** | **2** | **3** | **4** | **5** | **6** |
| L-tryptophan | C00078 | 3.14E+07 | 3.27E+07 | 3.17E+07 | 2.64E+07 | 3.03E+07 | 2.11E+07 |
| 5-Hydroxyindoleacetate | C05635 | 1.10E+05 | 1.32E+05 | 9.79E+04 | 1.01E+05 | 1.19E+05 | 8.31E+04 |
| 3-Methyleneoxindole | C02796 | 2.61E+05 | 2.79E+05 | 2.81E+05 | 2.42E+05 | 2.71E+05 | 1.87E+05 |
| Indole-3-acetaldehyde | C00637 | 1.52E+05 | 1.49E+05 | 1.68E+05 | 1.27E+05 | 1.36E+05 | 9.98E+04 |
| Indolepyruvate | C00331 | 2.99E+05 | 6.88E+05 | 2.72E+05 | 9.63E+05 | 3.17E+05 | 1.54E+05 |
| Indoxyl | C05658 | 6.70E+04 | 7.38E+04 | 7.61E+04 | 8.17E+04 | 6.93E+04 | 7.79E+04 |
| kynurenine | C00328 | 4.95E+05 | 5.69E+05 | 3.92E+05 | 4.54E+05 | 4.57E+05 | 3.47E+05 |
| N-formyl kynurenine | C02700 | 8.05E+04 | 9.73E+04 | 7.92E+04 | 8.77E+04 | 1.36E+05 | 7.85E+04 |
| Anthranilate | C00108 | 1.02E+06 | 9.48E+05 | 1.12E+06 | 1.62E+06 | 1.70E+06 | 1.35E+06 |
| Picolinic acid | C10164 | 1.38E+05 | 1.20E+05 | 1.42E+05 | 7.55E+04 | 5.97E+04 | 7.03E+04 |
| g-Oxalo-crotonate | C03453 | 1.14E+05 | 8.14E+04 | 8.33E+04 | 2.21E+05 | 2.15E+05 | 2.07E+05 |
| 8-Methoxykynurenate | C05830 | 2.61E+04 | 3.92E+04 | 3.96E+04 | 7.54E+03 | 3.73E+03 | 9.34E+03 |
| Indole | C00463 | 2.82E+04 | 3.74E+04 | 3.46E+04 | 2.34E+04 | 2.87E+04 | 1.65E+04 |
| Indole-3-acetate | C00954 | 6.08E+04 | 7.43E+04 | 6.84E+04 | 6.93E+04 | 6.81E+04 | 4.78E+04 |
| quinolinic acid | C03722 | 2.64E+05 | 3.37E+05 | 3.34E+05 | 1.96E+05 | 2.96E+05 | 2.22E+05 |
| 2-Aminomuconate | C02220 | 2.87E+05 | 2.23E+05 | 3.54E+05 | 1.43E+05 | 1.98E+05 | 1.43E+05 |
