# Supplementary material for: Targeting Tryptophan Catabolism in Ovarian Cancer to Attenuate Macrophage Infiltration and PD-L1 Expression
Source: Cancer Res Commun. 2024 Mar 18;4(3):822–33. doi: 10.1158/2767-9764.CRC-23-0513 (PMC10946310; doi:10.1158/2767-9764.CRC-23-0513)
Supplement: Supplementary Methods [file crc-23-0513-s10.docx]

**SUPPLEMENTAL METHODS**

Tryptophan Depletion: OVCAR3 or COV504 cells (3,000/well) were seeded in 96-well plates with tryptophan containing media or tryptophan depletion media. OVCAR3 - RPMI-1640 without TRP; Cat#50-190-8106, Fisher Scientific with 10% Dialysis FBS (Cat#A3382001, Thermofisher) and COV504 - DMEM/F12 without TRP; Cat#MBS653056, MyBioSource LLC with 10% Dialysis FBS. Cell culture media with TRP is standard RPMI-1640 or DMEM/F12. After 8 hrs, KYN (Cat#K3750-500MG, Sigma) was added and cell proliferation was monitored 7 days via Incucyte.
